# 

**Published:** 1875-02

**Authors:** 


### Table of Contents.

ORIGINAL COMMUNICATIONS.

DYSMENORRHŒA. *By John M. Johnson, M.D., of Atlanta, Ga.*........ .. 641
CLINICAL STUDIES with large Non-emetic Doses of IPECACUANHA: With a Contribution to the Therapeusis of Cholera. *By Alfred A. Woodhull, M.D., Asst. Surg. U. S. A.*........................................ 659

ATLANTA ACADEMY OF MEDICINE.

Case of Perineal Section—Coma and Convulsions following a hearty meal—Adulterated Ipecac—Atony of Bladder following concussion of a fall—Peritomy for Corneal Opacity—Case of supposed Gout—Case of Multiple Fractures—Treatment of Erysipelas—Discussion upon Syphilis—Discussion upon Dysmenorrhœa—Case of Epistaxis treated by Cording the Arms........................................ 673

OUR EXCHANGES.

Darwinism in the Kitchen........................................ 684
Normal Ovariotomy, Case, Recovery........................................ 685
Abortion, Treatment of........................................ 688
Scopemania........................................ 689
State Medicine........................................ 696
Electro-Medical Humbug........................................ 697
Quinine a Cardiac Sedative in Hemorrhage........................................ 698
Worthless Quinia Pills—Chloroform in Labor........................................ 699

BIBLIOGRAPHICAL.

Beard & Rockwell on Medical and Surgical use of Electricity........................................ 700
Dental Pathology and Surgery—*Salter*........................................ 701
Pamphlets........................................ 702

EDITORIAL.

Association Medical Officers Confederate States Army and Navy........
Toxicological use of Hypodermic Syringe........................................ 704

